# Machine Learning-Based Genome-Wide Association Study Reveals Genetic Loci Associated with Body Measurement Traits in Yili Horses

**DOI:** 10.3390/ani16091373

**Published:** 2026-04-29

**Authors:** Zhehong Shen, Liping Yang, Yuheng Xue, Xiaokang Chang, Jingxuan Shen, Weijun Sun, Yaqi Zeng, Jun Meng, Xinkui Yao

**Affiliations:** 1 College of Animal Science, Xinjiang Agricultural University, Urumqi 830052, China; 18858657690@163.com (Z.S.); yanglp98@126.com (L.Y.); xyh9523@126.com (Y.X.); changxk312@126.com (X.C.); 18914889860@163.com (J.S.); 15352590926@163.com (W.S.); junm86@xjau.edu.cn (J.M.); yxk61@126.com (X.Y.); 2 Xinjiang Key Laboratory of Horse Breeding and Exercise Physiology, Urumqi 830052, China

**Keywords:** machine learning, genome-wide association study, Yili horses, body measurement

## Abstract

Body measurement traits are key indicators for evaluating growth performance, production potential, and breeding value in Yili horses. However, studies investigating the association between body measurement traits and mutation loci in Yili horses remain limited. In this study, 255 adult Yili mares were used as the study population, including 152 speed-type and 103 meat-type individuals. Whole-genome resequencing was performed, and four phenotypic traits and body weight were measured. A mixed linear model (MLM)-based genome-wide association study (GWAS) was conducted using GEMMA (v 0.98.5), incorporating age, farm effects, and top three principal components as covariates. In parallel, a machine learning-based GWAS (ML-GWAS) framework integrating Lasso regression for feature selection and Random Forest (RF) with five-fold cross-validation was applied to improve the detection of complex genetic signals. Using both conventional GWAS methods and machine learning-based GWAS approaches, a total of 238 mutation loci significantly associated with body measurement traits were identified, and 277 candidate genes were annotated. These genes may play a role in several biological processes, including skeletal development, muscle formation, cell growth, energy metabolism, and protein synthesis. The findings suggest that genetic variations have already manifested among the studied groups. The results indicate that genetic differences have already emerged among different Yili horse populations at the genomic level. Furthermore, this study demonstrates that integrating machine learning with conventional GWAS effectively improves the detection efficiency of loci associated with complex traits, while also providing new molecular evidence for understanding the genetic mechanisms underlying differences in body measurement traits among Yili horse groups.

## Simple Summary

Body measurement traits are important traits because they influence athletic ability, production potential, and overall health. However, the genetic factors that control body measurement traits in Yili horses, an important horse breed in China, are still not fully understood. In this study, we analyzed genetic information from 255 Yili mares and measured four body measurement traits and body weight, including wither height, body length, heart girth, and cannon bone circumference. By combining traditional genetic association analysis with a computer method that can detect complex patterns in large datasets, we identified many regions in the horse genome that are related to body measurement traits. In total, we discovered 238 genetic variations and 277 potential genes that may influence growth and body structure. Several of these genes are known to be involved in processes such as bone development, muscle growth, energy metabolism, and cell growth, which are all important for shaping the body of a horse. Our results also show that combining traditional statistical methods with modern computer learning approaches can improve the ability to detect genes related to complex traits. These findings provide new knowledge about the biological mechanisms that control body measurement traits in horses and offer useful genetic information that may help breeders develop more efficient breeding strategies to improve horse performance and production.

## 1. Introduction

Body measurement traits are classic quantitative traits that have attracted continuous attention from geneticists for more than a century. These traits not only determine the external morphological characteristics of horses but also directly influence their athletic ability, production performance, and health status [1,2,3,4]. In recent years, studies based on resequencing data have successfully revealed the domestication history of horses and clarified the genetic mechanisms underlying traits such as coat color [5], gait [6], and athletic performance [7], thereby providing important theoretical support for genomic selection breeding. Previous studies have shown that key genes, such as LCORL, HMGA2, ZFAT, and NCAPG, account for approximately 83% of the variation in equine body conformation [8]. In addition, He et al. [9] reported that, in Yili horses, the SNP locus BIEC2-808543 located approximately 100 kb upstream of the LCORL gene was significantly associated with wither height, body length, heart girth, and cannon bone circumference.

However, conventional genome-wide association study (GWAS) approaches still have limitations when handling high-dimensional genomic data and complex genetic architectures, making it difficult to comprehensively capture loci with small effects or nonlinear genetic signals. Machine learning (ML) methods, owing to their strong pattern recognition capabilities and ability to process high-dimensional data, have been increasingly introduced into genetic association analyses, partially overcoming the limitations of traditional statistical models. Among these, the Random Forest (RF) algorithm proposed by Breiman [10] has become one of the most widely used methods in genomic studies due to its robustness and feature selection capability. Moreover, Yoosefzadeh-Najafabadi et al. [11] demonstrated that support vector regression (SVR)-mediated GWAS can complement conventional GWAS approaches and improve the detection power for complex traits.

In recent years, the modern equine industry has developed rapidly, and the role of horses has progressively expanded beyond traditional draft work to encompass horse racing, equestrian sports, and the production of specialized equine products. Therefore, the objective of this study was to integrate whole-genome sequencing data and to apply a machine learning-assisted GWAS framework to identify key genetic variants that are difficult to detect using conventional GWAS approaches. Furthermore, this study aimed to screen and characterize candidate genes significantly associated with body measurement traits, thereby providing new molecular insights into the genetic mechanisms underlying morphological variation in Yili horses and establishing a theoretical foundation for precision breeding based on genomic information.

## 2. Materials and Methods

### 2.1. Experimental Materials

A total of 255 adult Yili mares were selected from Yili Stud Farm Agricultural and Animal Husbandry Technology Co., Ltd. (Ili Kazakh Autonomous Prefecture, Xinjiang, China) and Zhaosu Horse Farm Agricultural and Animal Husbandry Development Co., Ltd. (Ili Kazakh Autonomous Prefecture, Xinjiang, China). The study population included 152 speed-type Yili mares (ST) and 103 meat-type Yili mares (MT). Only mares were included in this study because Yili horses are still undergoing crossbreeding improvement, in which local Yili mares serve as the maternal genetic foundation, while stallions are mainly derived from introduced breeds such as Thoroughbred and Ardennes horses. In addition, the studied populations represent core breeding mare groups from the two farms.

**Figure 1:**
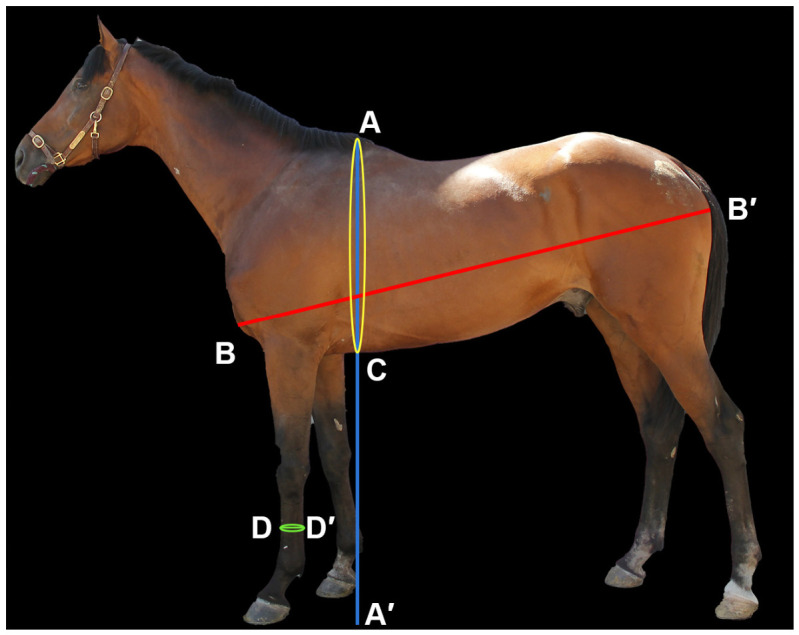
Schematic diagram of body measurement traits in horses. A–A′ represents wither height (WH), B–B′ represents body length (BL), A–C represents heart girth (HG), and D–D′ represents cannon bone circumference (CBC).

As shown in Figure 1, four body measurement traits and body weight (BW) were measured for each horse using a measuring tape, measuring stick, and weighbridge, including wither height (WH), body length (BL), heart girth (HG), and cannon bone circumference (CBC). Additionally, 5 mL of jugular venous blood was collected from each horse into EDTA tubes and stored at −20 °C until DNA extraction. Xinjiang Compson Biotechnology Co., Ltd. (Changji, Xinjiang, China) performed whole-genome resequencing.

### 2.2. DNA Extraction and Sequencing

Genomic DNA was extracted using the phenol–chloroform method, followed by quality assessment using agarose gel electrophoresis and quantification with the dsDNA HS Assay Kit on a Qubit 4 Fluorometer (Thermo Fisher Scientific, Waltham, MA, USA). High-quality DNA samples were randomly fragmented to the desired insert size (~350 bp) using ultrasonic shearing, and sequencing libraries were constructed according to the manufacturer’s standard protocols, including end repair, A-tailing, adapter ligation, and PCR amplification. Paired-end sequencing (150 bp) was performed on the DNBSEQ-T7 platform (MGI Tech, Shenzhen, China). This platform is based on DNA nanoball (DNB) technology and combinatorial Probe-Anchor Synthesis (cPAS).

### 2.3. Alignment and SNP Detection of Sequencing Data

Raw genomic sequence data were quality-filtered using fastp (v 0.23.4) [12] with the parameters “-w 4 -q 20 -n 10 -u 40”. Specifically, “-w 4” indicates the use of four processing threads to improve computational efficiency; “-q 20” sets the minimum Phred quality score threshold of 20 for base filtering, corresponding to a base call accuracy of 99%; “-n 10” removes reads containing more than 10 ambiguous bases (*N*); and “-u 40” discards reads in which more than 40% of bases have a quality score below the specified threshold. These parameters were applied to remove reads with excessive ambiguous bases, low-quality sequences, or adapter contamination, thereby ensuring high-quality clean data for downstream analysis. Clean reads were then mapped and aligned to the EquCab3.0 reference genome (https://ftp.ensembl.org/pub/release-113/fasta/equus_caballus/dna/Equus_caballus.EquCab3.0.dna.toplevel.fa.gz, accessed on 1 May 2025) using BWA (v 0.7.17) [13]. Quality control evaluation for each sample was calculated using Qualimap (v 2.2.1) [14]. Variant detection across all samples was performed using the HaplotypeCaller module in GATK (v 4.4.0) [15]. To improve the quality of variant detection, all raw SNPs were filtered using the “Variant Filtration” module in GATK with the following parameters: Quality by Depth (QD) < 2.0, Fisher Strand Bias (FS) > 60.0, Strand Odds Ratio (SOR) > 3.0, Mapping Quality (MQ) < 40.0, Mapping Quality Rank Sum Test (MQRankSum) < −12.5, QUAL < 30.0, and Read Position Rank Sum Test (ReadPosRankSum) < −8.0. These parameters are widely used quality metrics for variant filtering and are recommended in the GATK Best Practices guidelines.

### 2.4. SNP Quality Control

PLINK (v 1.9) software [16] was used to perform multi-step quality control on the raw data to screen for valid loci. First, individuals with a genotype call rate lower than 90% were removed using the parameter “--mind 0.1”. Second, SNPs were filtered based on a minor allele frequency (MAF) greater than 5% and a SNP missing rate lower than 10%, using the parameters “--maf 0.05 --geno 0.1 --chr-set 31”. To avoid potential biases of sex chromosomes in sex-related genetic structures and to improve the stability and accuracy of GWAS analysis, autosomal SNPs (1–31) were selected.

### 2.5. Population Structure Analysis

To examine the population’s genetic structure, we applied two complementary approaches: Neighbor-Joining (NJ) tree construction and principal component analysis (PCA). Pairwise genetic distance matrices were calculated with PLINK and converted to Newick format in R (v 4.5.1). The NJ tree was subsequently visualized using the ggtree (v 3.16.3) [17]. PCA was performed using PLINK (v 1.9) with the parameter “--pca 10”, and the resulting sample scatter plots were generated using scatterplot3d (v 0.3.45) [18]. Genetic differentiation between populations was estimated using the fixation index (Fst) calculated with VCFtools (v 0.1.16) [19] based on the Weir and Cockerham method, using the --weir-fst-pop option with predefined population groups (speed.lst and meat.lst).

### 2.6. Conventional Genome-Wide Association Study (GWAS)

GWAS analysis was performed using a mixed linear model (MLM) implemented in GEMMA (v 0.98.5) [20,21]. Age, farm effect, and the first three principal components derived from PCA were included in the model. The mixed linear model was defined as follows (Equation (1)):

(1) Y=SNP+PCs+kinship+e

where *Y* represents the phenotypic vector; *SNP* denotes the fixed effect vector; *PCs* represent the principal components used to correct for population structure; *kinship* is the kinship matrix; and *e* represents the random residual effect vector. The K matrix was calculated based on SNP markers to estimate genetic relatedness among individuals.

To reduce the increase in false positives caused by multiple testing, multiple corrections were applied to the GWAS results. The Bonferroni-corrected genome-wide significance threshold was set at 9.0 × 10^−8^ (1/11,074,706), and the suggestive significance threshold was set at 1 × 10^−6^. Finally, the GWAS results were visualized by generating Manhattan plots and quantile-quantile (Q-Q) plots using the CMplot (v 4.5.1) [22].

### 2.7. Machine Learning-Based GWAS (ML-GWAS)

#### 2.7.1. Lasso Regression for Feature SNP Selection

To reduce SNP dimensionality, eliminate redundant genetic markers, and alleviate multicollinearity, Least Absolute Shrinkage and Selection Operator (Lasso) regression implemented in the glmnet (v 4.1-10) [23] was applied to perform preliminary feature selection on the quality-controlled genome-wide SNP dataset. SNPs with non-zero regression coefficients were retained as the feature SNP set, thereby achieving dimensionality reduction in high-dimensional genetic markers and preliminary screening of key loci. Unlike conventional mixed linear models used in GWAS, Lasso is a penalized regression approach that performs variable selection and coefficient shrinkage simultaneously. The Lasso model was defined as follows (Equation (2)):

(2) $$J(\beta )\;=\sum_{i=1}^{n}(y_{i}-{\hat{y}}_{i}{)}^{2}\;+\;\lambda \sum_{\;j=1}^{\;p}\beta_{j}$$

where $y_{i}$ represents the target variable, ${\hat{y}}_{i}$ denotes the predicted value of the model, *β_j_* represents the regression coefficient, *λ* is the regularization hyperparameter controlling the shrinkage degree of the regression coefficients, and p represents the number of independent variables. The core of Lasso regression lies in the L1 penalty, which imposes an absolute-value constraint on the coefficients. This property allows some regression coefficients to shrink to zero, thereby enabling effective feature selection.

#### 2.7.2. Random Forest (RF)

Based on the feature SNPs selected by Lasso regression, the Random Forest (RF) algorithm implemented in the caret (v 7.0-1) [24] was applied to perform genome-wide association analysis (GWAS) and to evaluate the association strength between SNPs and the target traits. The Random Forest model was defined as follows (Equation (3)):

(3) $$Y_{i}\;=\;\frac{1}{B}\sum_{b=1}^{B}T_{b}(X_{i})$$

where *Y_i_* represents the predicted value for genotype *X_i_*, *T* denotes the prediction result of the constructed trees, and *B* represents the total number of trees in the forest. In this study, the ntree parameter was set to the default value of 500, and the mtry parameter was defined as the square root of the number of columns in x_train or one-third of the number of columns in x_train. The ML-GWAS analysis was conducted by measuring the importance of each feature through five-fold cross-validation [25], where each feature corresponded to an SNP in this study.

### 2.8. Gene Functional Annotation

Based on Ensembl annotations and using the Equus caballus reference genome EquCab 3.0, the 100 kb regions upstream and downstream of significant SNP loci were annotated using ANNOVAR (version 2020-06-08) [26]. The genes identified from the two GWAS methods were intersected, and an upset plot was generated using the R package UpSetR (v 1.40) to visualize the overlapping gene sets.

### 2.9. Phenotypic Data Analysis

One-way analysis of variance (ANOVA) was performed using SPSS 26.0 to evaluate differences in four body measurement traits and body weight between the two Yili horse groups. Differences were considered extremely significant at *p* < 0.01.

## 3. Results

### 3.1. Descriptive Statistics of Phenotypes

In this study, four body measurement traits and body weight of 255 Yili horses were analyzed. For each trait, the mean, standard deviation (SD), maximum (Max), minimum (Min), and coefficient of variation (CV) were calculated (Table 1). The CV for body weight (BW) was the highest at 11.17% and 14.37%, followed by cannon bone circumference (CBC) and heart girth (HG), while the CV for wither height (WH) was the lowest at 2.05% and 3.40%. The results of the ANOVA showed that the mean values of WH, BL, and BW in speed-type Yili horses were extremely significantly higher (*p* < 0.01) than in meat-type Yili horses, whereas the mean cannon bone circumference was extremely significantly lower (*p* < 0.01). No significant difference was observed in HG between the two groups. These results indicate that the study population exhibits abundant genetic variation in body measurement traits, providing substantial potential for selection and a solid data foundation for subsequent genome-wide association analyses.

### 3.2. Genome Resequencing and Identification of SNPS

**Figure 2:**
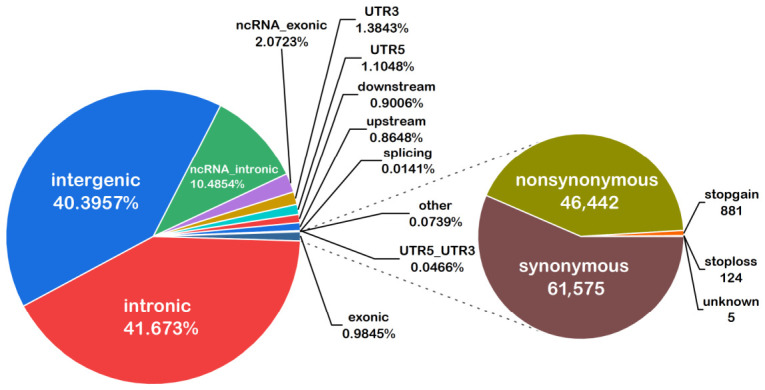
Functional distribution of SNPs in Yili horse. The pie chart to the left shows the distribution of SNPs across intergenic, intronic, upstream, downstream, UTR3, UTR5, splicing, ncRNA, and exonic regions. The pie chart to the right displays the breakdown of exons, including nonsynonymous, synonymous, stop-gain, and stop-loss exons.

**Figure 3:**
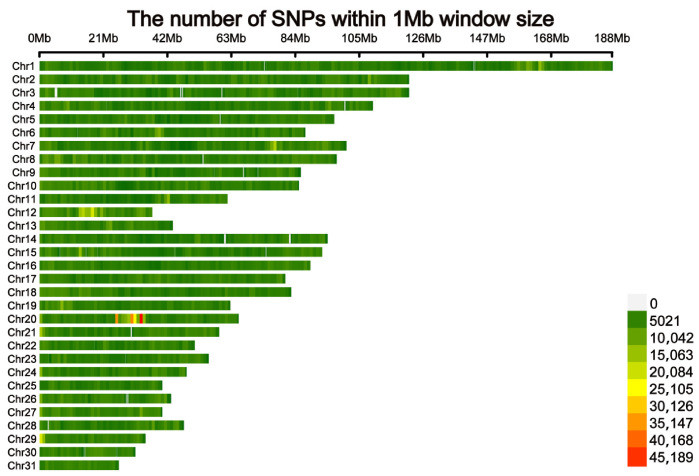
Distribution of SNPs on each autosome chromosome.

Whole-genome resequencing was performed on blood samples from 255 Yili horses. The total sequencing data amounted to 8475.83 Gb, with an average GC content of 43.64%. All samples were aligned to the EquCab3.0 reference genome, achieving an average mapping rate of 99.92% and an average sequencing depth of 13.25×, meeting the quality requirements (Supplementary Table S1). Following variant detection and quality control, a total of 11,074,706 SNPs were obtained and retained for further post-QC association analyses. SNPs located within intronic and intergenic regions accounted for 82.07% of the total, while the remaining variants were distributed across exons, untranslated regions (UTRs), and splice sites (Figure 2). The distribution of SNPs on each autosome chromosome is shown in Figure 3.

### 3.3. Population Genetic Structure

**Figure 4:**
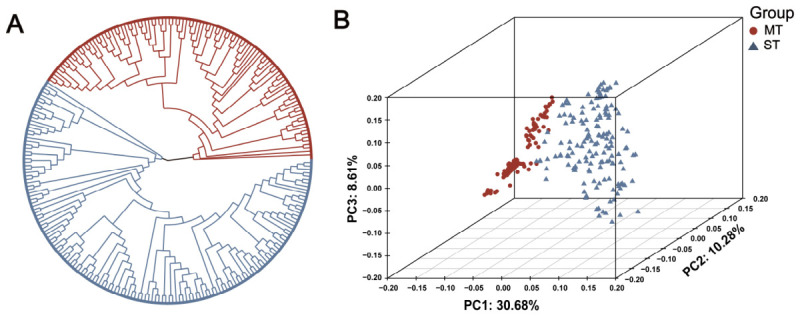
Population genetics analyses of samples. (**A**) Phylogenetic trees of two types of Yili horse populations were constructed based on the neighbor-joining method; (**B**) PCA results for two types of Yili horse populations.

Based on the quality-controlled VCF file data, the genetic relationships between the two Yili horse populations were analyzed. The NJ-tree was constructed using individual SNP variations and showed that MT and ST were clearly separated into two distinct genetic clusters (Figure 4A). In addition, the first principal component (PC1) and the second principal component (PC2) explained 30.68% and 10.28% of the total genetic variation, respectively, further supporting the genetic differentiation between the two populations and clearly distinguishing them into the MT and ST groups (Figure 4B and Supplementary Table S2). The weighted F_ST value estimated using the Weir and Cockerham method was 0.051189, indicating a low to moderate level of genetic differentiation between the two populations. This suggests that different types of Yili horses are still in an ongoing stage of improvement toward specialized breeding, with genetic differentiation gradually emerging during the selection process.

### 3.4. GWAS Analysis of Body Measurement Traits and Candidate Genes

#### 3.4.1. Conventional GWAS Analysis

**Figure 5:**
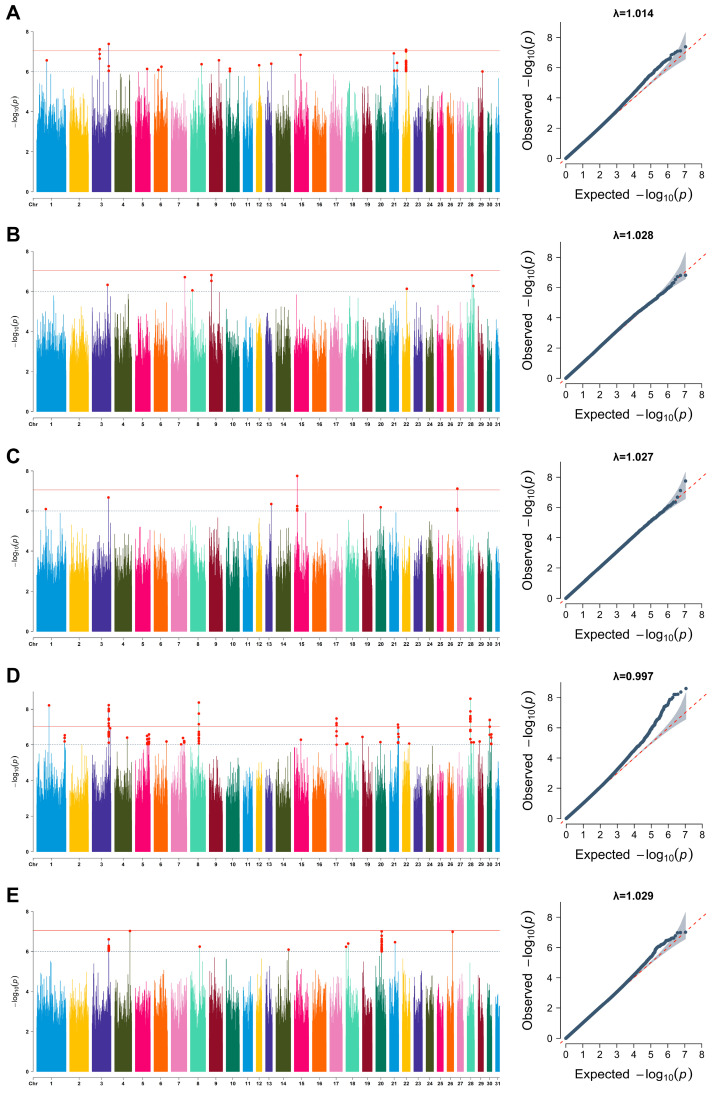
Manhattan plot and Q-Q plot of genome-wide association study for (**A**) wither height; (**B**) body length; (**C**) heart girth; (**D**) cannon bone circumference; and (**E**) body weight. The horizontal solid lines and dashed lines denote the genome-wide significance (9.0 × 10^−8^) and suggestive significance thresholds (1.0 × 10^−6^), respectively. The red dots are SNPs that exceeded the suggestive significance thresholds. The blue dots in Q-Q plots represent the observed *p*-values for each SNP. The gray shadow in the Q-Q plots indicates the confidence interval. The diagonal dashed line in Q-Q plots indicates the expected distribution under the null hypothesis of no association.

Principal components 1–3 (PCs 1–3), age, and breed were included as covariates in the mixed linear model (MLM) to perform GWAS analysis of body measurement traits. The results are shown in Figure 5 (Manhattan and Q-Q plots). The genomic inflation factor (λ) ranged from 0.997 to 1.029. The Q-Q plots indicated a good agreement between the observed and expected distributions, implying that the effects of population structure and genetic background were adequately accounted for and that the association results were dependable. Using a suggestive significance threshold of *p* < 1.0 × 10^−6^, a total of 210 significant SNPs were identified, which were annotated to 207 candidate genes highly associated with body measurement traits (Table 2 and Supplementary Table S3). Among these, 31 SNPs reached the more stringent genome-wide significance level of *p* < 9.0 × 10^−8^. These 31 SNPs were significantly associated with wither height, heart girth, and cannon bone circumference, with 10 loci primarily concentrated on chromosome 3.

#### 3.4.2. Machine Learning-Based GWAS

**Figure 6:**
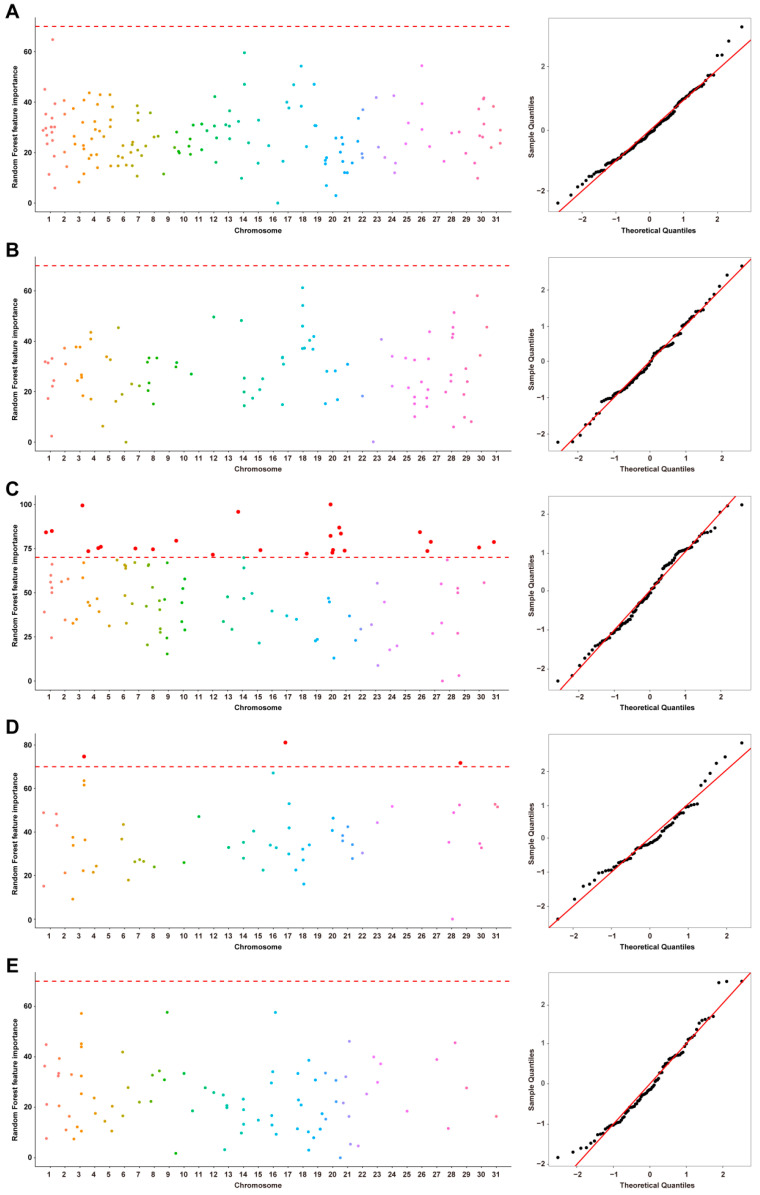
Manhattan plot and Q-Q plot of machine learning-based genome-wide association study for (**A**) wither height; (**B**) body length; (**C**) heart girth; (**D**) cannon bone circumference; and (**E**) body weight. The horizontal dashed lines denote the suggestive random forest feature importance (70%). The red dots indicate SNPs that exceeded the suggestive threshold for random forest feature importance. The blue dots in Q-Q plots represent the observed values for each SNP. The diagonal solid line in Q-Q plots indicates the expected distribution under the null hypothesis of no association.

Genome-wide association analyses of body measurement traits were performed using the Random Forest (RF) machine learning model, with results shown in Figure 6. The Q-Q plot indicated that the observed distribution of feature importance largely aligned with the expected distribution, with only slight deviations in the upper quantiles, suggesting that the model fit was generally good, systematic bias was minimal, and loci with high importance were more likely to reflect true genetic signals. The five-fold cross-validation results showed that the models performed well across all traits. The average R^2^ values ranged from 0.708 to 0.838, while the RMSE values varied depending on the trait. Overall, the relatively stable performance across folds suggests that the models have good robustness and are not overfitted (Supplementary Tables S4–S8). Using a feature importance threshold greater than 70%, 28 significant SNPs were identified, which were annotated to 80 candidate genes highly associated with heart girth (ML_HG) and cannon bone circumference (ML_CBC), with detailed results provided in Table 3.

### 3.5. Genetic Overlap and Specificity of Body Measurement Traits Between Different Analytical Methods

**Figure 7:**
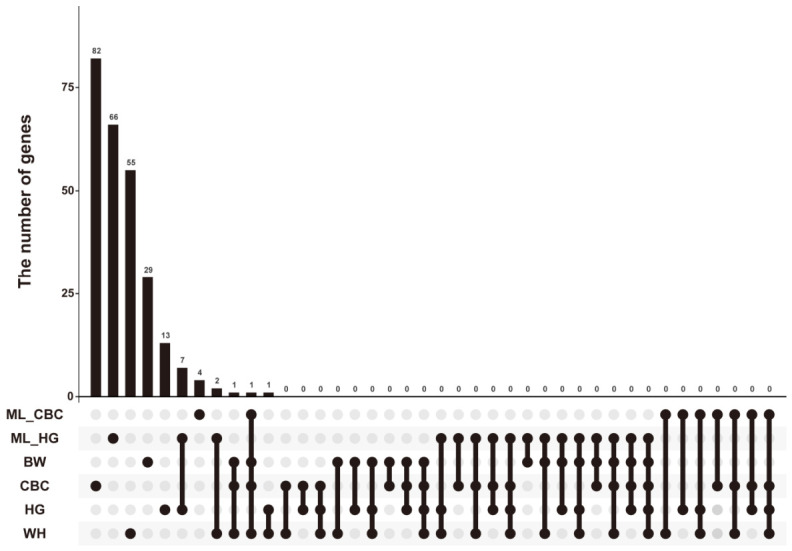
Upset plot diagram of candidate genes for each trait of conventional GWAS and ML-GWAS. The connected dots below the bar plot illustrate the specific comparisons for each intersection. The bar plot at the top shows the intersection size. ML_CBC: Machine-Learning based cannon bone circumference; ML_HG: Machine-Learning based heart girth; BW: body weight; CBC: cannon bone circumference; HG: heart girth; WH: wither height.

Using candidate gene sets derived from six growth-related phenotypes (ML_CBC, ML_HG, BW, CBC, HG, and WH), an upset plot was constructed to evaluate the extent of genetic sharing among traits between the two analytical approaches (Figure 7). The results revealed substantial overlap among candidate genes across the different growth phenotypes, with 10 genes consistently annotated by both GWAS methods, suggesting that these genes represent core genetic components regulating growth traits. At the inter-phenotype genetic overlap level, seven genes associated with the HG phenotype were consistently identified by both GWAS approaches. Additionally, one highly overlapping candidate gene was identified between body weight (BW), cannon bone circumference (CBC), and wither height (WH) detected by traditional GWAS and the cannon bone circumference trait identified by ML-GWAS (ML_CBC). This finding indicates that certain body measurement traits may be regulated by common growth and developmental pathways. Moreover, a substantial number of genes were shared between the candidate gene set identified via machine learning and those from traditional phenotype-based association analyses, confirming the reliability and effectiveness of machine learning methods in capturing biologically relevant genetic signals from high-dimensional SNP data.

## 4. Discussion

Regarding the background of Yili horses, the breed originated in Zhaosu County, Ili Kazakh Autonomous Prefecture, Xinjiang, China. It was developed in the 1980s through crossbreeding between local Kazakh mares and stallions from breeds such as Orlov, Budyonny, and Don. In 1985, it was officially recognized as a new cultivated breed. With the development of the modern horse industry in China, Yili horses have undergone further selective breeding for specialized purposes. The two groups identified in this study were formed through subsequent improvement processes, in which Yili horses were used as the maternal base and crossbred with Thoroughbred or Ardennes horses. The result of ANOVA, population-genetic structure analysis and the weighted F_ST value supported the division of Yili Speed Type and Meat Type horses into two distinct genetic clusters. These findings indicate that selective breeding using different sires has already led to differentiation between the two types, establishing a stable genetic foundation. Therefore, they further demonstrate that systematic crossbreeding can generate new populations with independent genetic structures through recombination, providing valuable insights for breeding strategies targeting specialized-purpose Yili horses.

GWAS has been widely used to identify candidate genes and genomic regions associated with diverse traits. For example, previous GWAS studies have identified key loci and genes associated with production and reproductive traits in chickens [27]. Accordingly, in this study, we aimed to apply both conventional GWAS and machine learning-based GWAS (ML-GWAS) to analyze body measurement traits in Yili horses to identify associated genetic loci and key candidate genes comprehensively. Using the two GWAS approaches, a total of 238 significantly associated SNPs were identified. Annotation of these significant loci yielded 277 candidate genes, of which both methods consistently identified 10. These candidate genes provide crucial insights into the molecular regulatory mechanisms underlying body measurement traits in Yili horses. Notably, well-characterized candidate genes such as *KCNIP4*, *PPA1*, *EIF3H* and *LRRC20* may collectively contribute to body measurement trait formation by regulating multiple biological processes, including skeletal development, muscle formation, cell proliferation, energy metabolism, and neuromuscular signaling.

Potassium Voltage-Gated Channel Interacting Protein 4 (*KCNIP4*) is a gene encoding a member of the family of voltage-gated potassium (Kv) channel-interacting proteins (KCNIPs), which belong to the recoverin branch of the EF-hand superfamily. It plays key physiological roles in facilitating neurotransmitter release, smooth muscle contraction, heart rate regulation, and insulin secretion [28]. The *KCNIP4* gene has been associated with growth traits in broilers [29,30], sheep [31,32], and beef cattle [33,34]. Potassium channel regulatory factors not only influence neural signal transmission but also, during short-term high-intensity exercise, enhance the body’s load capacity by modulating neurotransmitter release, accelerating heart rate, stimulating insulin secretion, and increasing neuronal excitability. They can also indirectly affect body measurement trait development by regulating muscle contraction efficiency and physical performance [35]. In the present study, *KCNIP4* was identified as a candidate gene associated with body measurement traits in Yili horses. Notably, previous GWAS analyses in Yili racehorses have also highlighted *KCNIP4* as a potential locus influencing ranking performance [7]. However, the functional interpretation of *KCNIP4* may differ between utilization types. For speed-type Yili horses, *KCNIP4* may contribute to enhanced neuromuscular coordination, rapid signal transmission, and cardiovascular responsiveness, which are critical for high-intensity exercise performance and may indirectly influence body traits such as heart girth. In contrast, in meat-type Yili horses, its role may be more closely related to metabolic regulation, muscle contraction efficiency, and energy utilization, thereby potentially affecting muscle development and body measurement traits.

Inorganic pyrophosphatase 1 (*PPA1*) is a member of the inorganic pyrophosphatase (PPase) family, whose primary function is to catalyze the hydrolysis of pyrophosphate, thereby generating energy to facilitate biosynthetic reactions. Eukaryotic translation initiation factor 3 (EIF3) is a large multiprotein complex that initiates protein translation by forming a complex with ribosomes and mRNA [36]. *EIF3H*, an essential subunit of the EIF3 family, promotes the reinitiation of translation by ribosomes, partially relieving inhibition of specific open reading frames [37]. Mutants of *EIF3H* in plants also exhibit auxin-related phenotypes [38]. Experimental reduction in the translation initiation factor eIF6 in mice results in decreased body weight and impaired tissue growth, due to reduced cell proliferation, indicating that initiation factors can directly influence growth characteristics [39]. Upregulation of *PPA1* [40] and *EIF3H* [41] has been associated with enhanced cell proliferation, survival, and metabolism in various human malignancies.

Additionally, *PPA1* has been identified as a key upstream regulator of adipogenesis, controlling adipose tissue development and systemic metabolic homeostasis [42]. In livestock, growth and development rely not only on continuous skeletal growth but also on the proliferation and differentiation of skeletal muscle cells. In this study, *PPA1* and *EIF3H* were significantly associated with body measurement traits, suggesting that they may influence skeletal and muscle cell proliferation and differentiation by modulating cellular metabolic efficiency, growth rate, and protein synthetic capacity, thereby playing a critical role in regulating body measurement traits.

Leucine-rich repeat containing 20 (*LRRC20*) belongs to the leucine-rich repeat protein family, which is typically involved in protein–protein interactions, cellular signaling, and structural regulation [43]. The *LRRC20* gene is expressed at relatively high levels in multiple tissues, particularly in the brain, testes, and certain smooth muscle-rich tissues; notably, its expression is even higher in skeletal muscle, where it also contains a greater number of intragenic enhancer elements [44]. In transcriptomic and proteomic studies of the longissimus dorsi muscle in Wannan Hu pigs, seven genes were significantly upregulated, including *LRRC20*, suggesting that this gene may play important roles in muscle development and fat deposition [45]. Although functional studies of *LRRC20* in horses are limited, evidence from other livestock species indicates that it is closely associated with skeletal muscle growth and development. Therefore, it is plausible that *LRRC20* participates in the regulation of skeletal or muscular structure, thereby influencing body measurement traits.

A candidate gene associated with body weight, ENSECAG00000033069, was identified in previous studies on horse meat [46]. In the present study, this gene was found to be highly associated with wither height, cannon bone circumference, and body weight. However, research on this gene remains limited, and its functional role has yet to be fully validated.

Although the ML models showed certain advantages in capturing complex patterns, no significant loci were detected for some traits. This may be mainly due to the limited sample size, which reduces the power to identify stable genetic signals. In addition, body measurement traits are typically controlled by multiple loci with small effects, making them difficult to detect under high-dimensional conditions. It should also be noted that ML methods are primarily developed for prediction rather than statistical inference and therefore may have limited ability to identify statistically significant loci compared with conventional GWAS approaches. Moreover, potential influences such as phenotypic measurement errors, population structure, and environmental factors may further obscure true associations. Taken together, the lack of significant loci for some traits does not necessarily indicate the absence of genetic effects, but rather reflects the limitations of the current data and analytical methods.

## 5. Conclusions

Analyzing the genetic architecture of body measurement traits in Yili horses using multiple approaches can help breeders develop more efficient breeding strategies. This study demonstrates that Random Forest (RF)-mediated GWAS can serve as a complementary tool to conventional GWAS, offering potential for dissecting complex traits and identifying specific loci in depth. By integrating the results from both conventional GWAS and ML-GWAS, a total of 238 significant SNPs and 277 key candidate genes were identified, of which 11 genes, such as *LRRC20*, *KCNIP4*, *PPA1*, and *EIF3H*, were overlapped in the two methods. These findings further underscore the effectiveness and feasibility of RF-mediated GWAS. In addition, the significantly associated loci and candidate genes identified in this study can be used as molecular markers for marker-assisted selection (MAS), enabling breeders to screen individuals with desirable genetic potential at an early stage. In addition, these loci can be incorporated into genomic selection (GS) models to improve the accuracy of estimated breeding values (EBVs) for body measurement traits.

## Figures and Tables

**Table 1:** Descriptive statistics of body measurement traits in different types of Yili horses.

Trait	Breed	*N*	Mean	Min	Max	SD	CV
WH	ST	152	155.26 ^A^	145	165	3.18	2.05%
	MT	103	144.34 ^B^	133.5	161.5	4.91	3.40%
BL	ST	152	156.02 ^A^	140	167	5.02	3.22%
	MT	103	144.92 ^B^	135	165	5.52	3.81%
HG	ST	152	176.85	159	190	6.56	3.71%
	MT	103	176.05	161	198	8.06	4.58%
CBC	ST	152	18.84 ^B^	16	21	0.68	3.61%
	MT	103	19.57 ^A^	17	24	1.84	9.41%
BW	ST	152	429.05 ^A^	315	564	47.94	11.17%
	MT	103	401.27 ^B^	317	556	57.66	14.37%

Note: Mean values with different capital letter superscript indicate extremely significant differences (*p* < 0.01), whereas values without letter superscripts indicate no significant difference (*p* > 0.05). WH: wither height; BL: body length; HG: heart girth; CBC: cannon bone circumference; BW: body weight.

**Table 2:** Data on significant loci for body confirmation traits in conventional GWAS.

Trait	Chr	Position	MAF	*p*-Value	Gene
WH	3	106,526,666	0.105	4.09 × 10^−8^	ENSECAG00000033069
WH	3	48,578,372	0.059	7.57 × 10^−8^	*CCSER1*
WH	22	24,358,461	0.089	8.01 × 10^−8^	*BPIFB6*; *BPIFB3*; *BPIFB4*; *SUN5*; *BPIFB2*; ENSECAG00000026847
HG	15	18,842,811	0.119	1.77 × 10^−8^	*CHMP3*; *KDM3A*; *REEP1*
HG	27	773,915	0.111	7.77 × 10^−8^	ENSECAG00000055124
CBC	28	20,803,808	0.156	2.52 × 10^−9^	*CEP83*; *TMCC3*
CBC	8	54,107,775	0.055	4.27 × 10^−9^	ENSECAG00000031630
CBC	3	106,594,561	0.126	5.96 × 10^−9^	ENSECAG00000033069; ENSECAG00000053647
CBC	1	79,035,569	0.053	6.17 × 10^−9^	ENSECAG00000009930; ENSECAG00000023376
CBC	1	79,035,596	0.053	6.17 × 10^−9^	ENSECAG00000009930; ENSECAG00000023376
CBC	3	106,594,813	0.126	9.67 × 10^−9^	ENSECAG00000033069; ENSECAG00000053647
CBC	3	106,594,238	0.128	1.22 × 10^−8^	ENSECAG00000033069; ENSECAG00000053647
CBC	3	106,624,869	0.134	1.29 × 10^−8^	ENSECAG00000033069; ENSECAG00000053647
CBC	28	20,817,030	0.136	1.33 × 10^−8^	*CEP83*; *TMCC3*
CBC	8	54,111,660	0.057	1.78 × 10^−8^	ENSECAG00000031630
CBC	28	20,783,685	0.140	2.46 × 10^−8^	*CEP83*; *TMCC3*
CBC	28	20,813,727	0.136	3.15 × 10^−8^	*CEP83*; *TMCC3*
CBC	17	43,170,064	0.055	3.34 × 10^−8^	ENSECAG00000051361; ENSECAG00000057773; ENSECAG00000060239
CBC	28	20,808,312	0.150	3.40 × 10^−8^	*CEP83*; *TMCC3*
CBC	3	106,623,924	0.128	3.42 × 10^−8^	ENSECAG00000033069; ENSECAG00000053647
CBC	28	20,803,092	0.156	3.85 × 10^−8^	*CEP83*; *TMCC3*
CBC	3	106,609,056	0.134	3.95 × 10^−8^	ENSECAG00000033069; ENSECAG00000053647
CBC	30	17,024,060	0.117	3.97 × 10^−8^	*USH2A*
CBC	30	17,039,544	0.101	4.08 × 10^−8^	*USH2A*
CBC	28	20,819,246	0.132	4.88 × 10^−8^	*CEP83*; *TMCC3*
CBC	17	43,156,103	0.059	6.18 × 10^−8^	ENSECAG00000057773; ENSECAG00000060239
CBC	3	106,597,776	0.136	6.29 × 10^−8^	ENSECAG00000033069; ENSECAG00000053647
CBC	8	54,148,562	0.055	6.89 × 10^−8^	ENSECAG00000031630
CBC	21	54,056,805	0.067	7.29 × 10^−8^	*MED10*; *ICE1*
CBC	17	43,115,042	0.067	7.95 × 10^−8^	ENSECAG00000057773; ENSECAG00000060239
CBC	3	106,646,418	0.130	8.80 × 10^−8^	ENSECAG00000033069; ENSECAG00000053647

Note: WH: wither height; HG: heart girth; CBC: cannon bone circumference.

**Table 3:** Data on suggestive importance loci for body confirmation traits in ML-GWAS.

Trait	Chr	Position	MAF	Importance	Gene
ML_HG	20	36,877,262	0.471	100.00	*SLC26A8*; *MAPK14*; *SRPK1*
ML_HG	3	104,664,666	0.082	99.434	*KCNIP4*
ML_HG	14	28,813,828	0.312	95.824	ENSECAG00000028172; ENSECAG00000037084; ENSECAG00000039703; ENSECAG00000043180; ENSECAG00000044153
ML_HG	21	1,910,461	0.114	86.896	ENSECAG00000019543; ENSECAG00000046973; ENSECAG00000058777
ML_HG	1	162,537,679	0.073	84.955	ENSECAG00000000419; *DAD1*; *ABHD4*; ENSECAG00000028590; ENSECAG00000032324; ENSECAG00000035225; ENSECAG00000057927
ML_HG	26	9,862,328	0.292	84.312	ENSECAG00000030912; *ROBO1*
ML_HG	1	58,750,435	0.055	84.200	*AIFM2*; *TYSND1*; *SAR1A*; *NPFFR1*; *LRRC20*; *PPA1*; *MACROH2A2*; ENSECAG00000047322; ENSECAG00000051984; ENSECAG00000059469
ML_HG	21	6,664,894	0.135	83.504	ENSECAG00000043830; ENSECAG00000057841; ENSECAG00000059141
ML_HG	20	36,803,130	0.410	82.206	*SLC26A8*; *CLPS*; *LHFPL5*; *SRPK1*; ENSECAG00000043440
ML_HG	9	62,131,512	0.326	79.454	*EIF3H*; ENSECAG00000050664
ML_HG	27	8,788,667	0.057	78.823	*KCNU1*
ML_HG	31	2,295,746	0.101	78.717	*KIF25*; *FRMD1*
ML_HG	4	98,000,855	0.426	76.011	*TPK1*
ML_HG	30	7,660,322	0.135	75.627	*ACBD3*; *LIN9*; ENSECAG00000033929
ML_HG	4	79,886,987	0.116	75.275	ENSECAG00000006212; ENSECAG00000023576; *HYAL4*
ML_HG	7	14,277,004	0.094	75.064	*DDI1*; *PDGFD*
ML_HG	7	77,779,271	0.059	74.595	*DNHD1*; *RRP8*; *ILK*; *TAF10*; *TPP1*; *DCHS1*; *MRPL17*
ML_HG	20	50,161,763	0.155	74.214	ENSECAG00000052318; ENSECAG00000047801
ML_HG	15	91,484,030	0.073	74.106	*PXDN*; *TPO*
ML_HG	21	17,356,675	0.053	73.842	*SLC38A9*; ENSECAG00000023296
ML_HG	27	60,238	0.067	73.612	ENSECAG00000046191
ML_HG	4	8,761,213	0.137	73.518	*STARD3NL*; ENSECAG00000019999; ENSECAG00000047697
ML_HG	20	46,598,412	0.053	72.685	*TNFRSF21*
ML_HG	18	72,330,147	0.116	72.146	*ANKRD44*; *CCDC150*; *GTF3C3*; *PGAP1*; ENSECAG00000058124
ML_HG	12	18,702,888	0.163	71.548	ENSECAG00000049092
ML_CBC	17	44,630,665	0.494	81.123	ENSECAG00000040338; ENSECAG00000045457
ML_CBC	3	106,786,407	0.102	74.686	ENSECAG00000053647
ML_CBC	29	1,189,636	0.069	71.742	ENSECAG00000046065; ENSECAG00000046786

Note: ML_HG: Machine-Learning based heart girth; ML_CBC: Machine-Learning based cannon bone circumference.

## Data Availability

The data and materials used in this research are available from the corresponding author on request.

## Supplementary Materials

The following supporting information can be downloaded at: https://www.mdpi.com/article/10.3390/ani16091373/s1, Table S1: Statistics of sequencing data evaluation; Table S2: The first three PCs of 255 Yili horses; Table S3: Data on suggested significant loci for body measurement trait; Table S4: Five-fold cross-validation results of WH; Table S5: Five-fold cross-validation results of BL; Table S6: Five-fold cross-validation results of HG; Table S7: Five-fold cross-validation results of CBC; Table S8: Five-fold cross-validation results of BW.

## Abbreviations

The following abbreviations are used in this manuscript:

GWAS	Genome-Wide Association Study
ML-GWAS	Machine Learning-Based Genome-Wide Association Study
ST	Speed-Type
MT	Seat-Type
WH	Wither Height
BL	Body Length
HG	Heart Girth
CBC	Cannon Bone Circumference
BW	Body Weight
